# Supplementary material for: Transcriptomics of Hirschsprung disease patient-derived enteric neural crest cells reveals a role for oxidative phosphorylation
Source: Nat Commun. 2023 Apr 15;14:2157. doi: 10.1038/s41467-023-37928-5 (PMC10105741; doi:10.1038/s41467-023-37928-5)
Supplement: Supplementary file 1 — Supplementary information [file 41467_2023_37928_MOESM1_ESM.pdf]

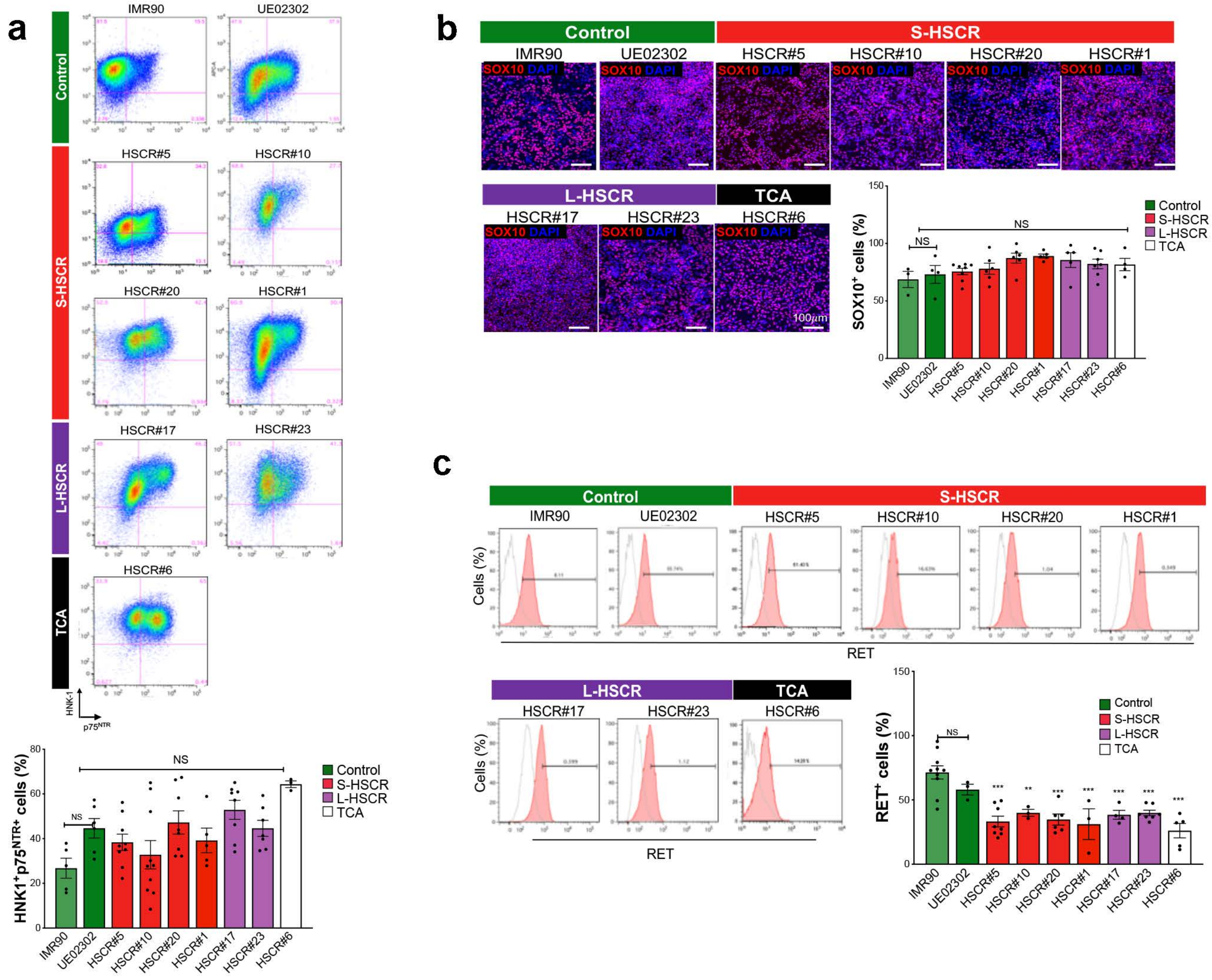

Supplementary Fig. 1 Characterization of hENCCs derived from control and HSCR-iPSC lines. Flow cytometry analyses of **(a)** HNK1^{+}p75^{NTR+} hENCCs.**(b)** Immunocytochemistry to detect SOX10 expression in HNK1/p75^{NTR} enriched hENCCs. **(c)** Flow cytometry analyses of RET expression in HNK1^{+}p75^{NTR+} ENCCs. Charts show the mean values +/- SEM from 3-10 independent experiments. *** and ** represent they are significantly different from the two control of *P*-value <0.001 and <0.01, respectively. NS: not significantly different (2-way ANOVA, Dunnett post test). Source data are provided as a Source Data file.

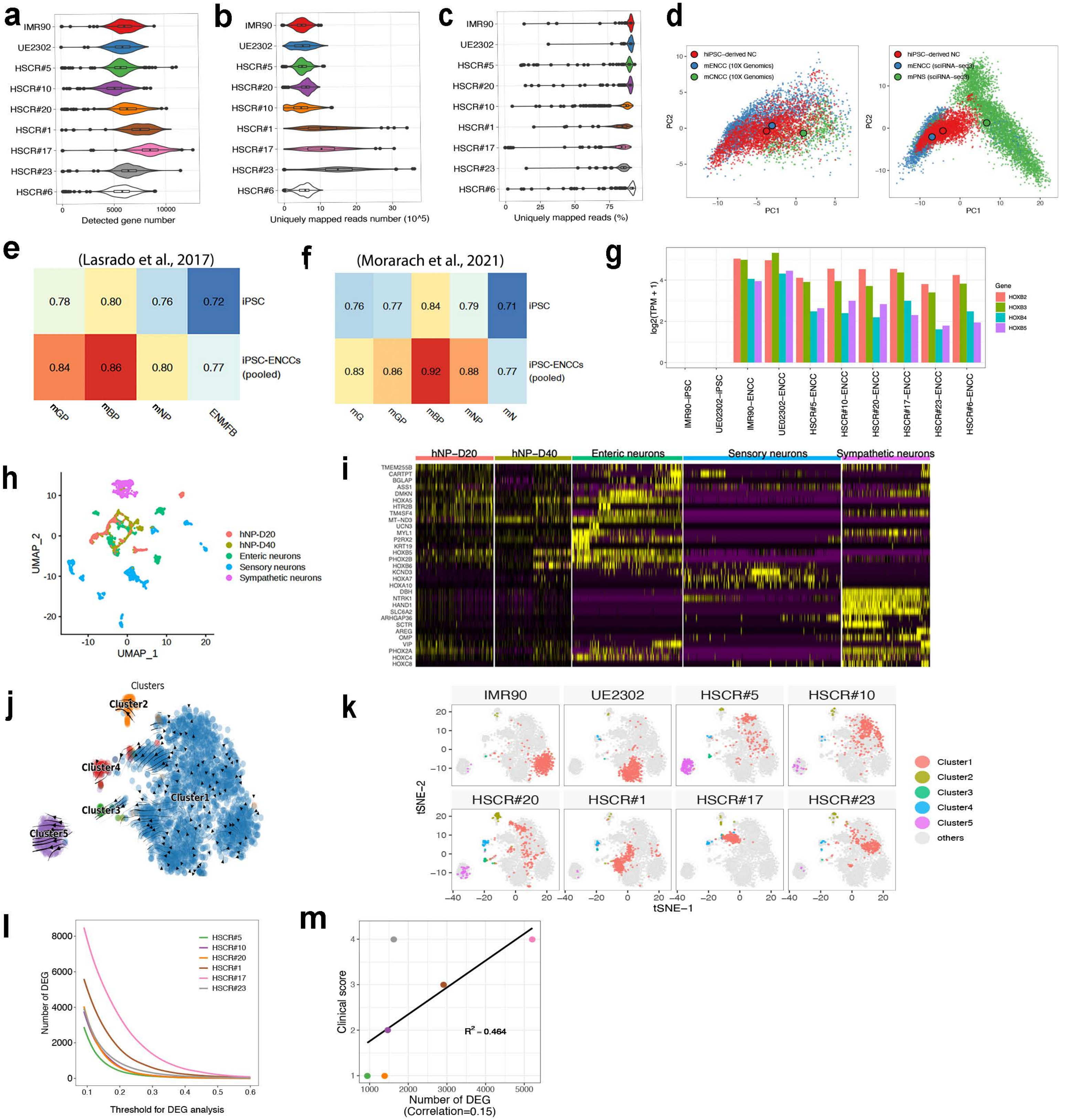

Supplementary Fig. 2 Quality control and overall characteristics of single-cell RNA-seq dataset. **(a)** The numbers of detected genes, **(b)** uniquely mapped reads and **(c)** the percentages of uniquely mapped reads of eight sequenced samples. **(d)** PCA plot shows the similarities between human iPSC-derived NC cells and mouse *in vivo* enteric/non-enteric NC cells at E13.5 from two independent datasets. Colored by cell group. Circled dots with bigger size indicate the average values of each cell group. Sequencing platforms of datasets are noted. Correlation heatmaps show the similarities between hiPSC, iPSC-derived ENCCs, and mouse enteric bipotent progenitors (mBP), glial progenitor (mGP), and mature glial cells (mG), neuronal progenitors (mNP), mature neurons (mN) and enteric mesothelial fibroblast (ENMFB) at **(e)** E12.5-13[21] and **(f)** E15.5, E18.5 and P21[24]. **(g)** Expression of *HOXB2, HOXB3, HOXB4, HOXB5* in ENCCs derived from the control and HSCR-iPSC lines based on the bulk RNAseq data. **(h)** UMAP analysis comparing the neuronal derivatives of hiPSC-ENCCs (hNP-D20, hNP-4) with mouse enteric, sensory and sympathetic neurons. **(i)** Heatmap shows the expression of marker genes in the neuronal derivatives of hiPSC-ENCCs (hNP-D20, hNP-4), mouse enteric, sensory and sympathetic neurons, and reveals that hiPSC-ENCCs highly similar to the mouse enteric neurons. **(j)** Predicted differentiation direction by RNA velocity. **(k)** Distribution of the cells from each sample in *t*-SNE plot. **(l)** Changes of numbers of DEGs at different thresholds in different HSCR-ENCCs. **(m)** Correlation between the number of DEG and clinical score among the six HSCR samples. R squared (R2) between number of DEGs and clinical score is calculated as the square of their correlation. In all the violin plots, each violin represents the kernel probability density of the data at different values. An inner boxplot indicates the interquartile range (IQR, the range between the 25th and 75th percentile) with the mid-point of the data.

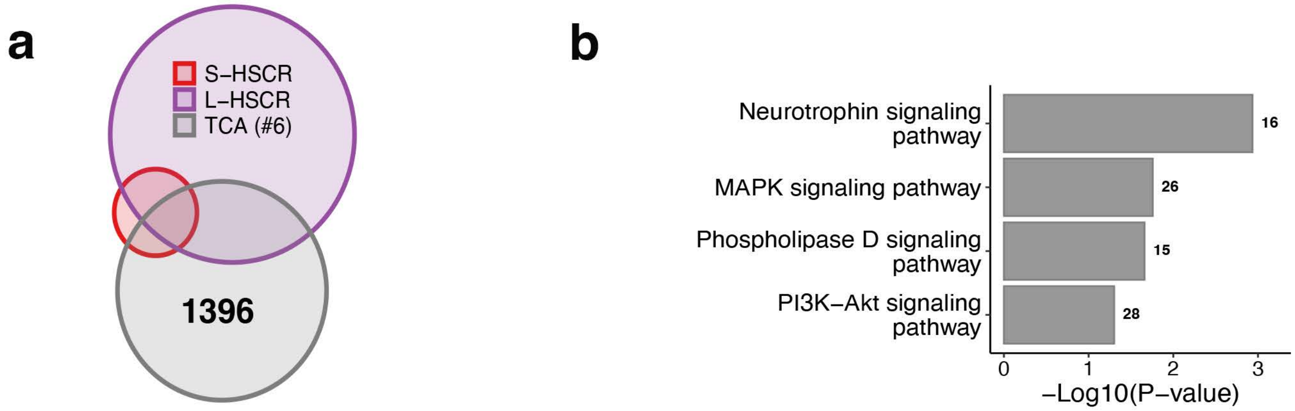

Supplementary Fig. 3 DEGs and pathways uniquely disrupted in TCA-ENCCs. (a) Venn plot shows the 1396 unique DEGs in TCA-ENCCs (HSCR#6) compared to S- and L-HSCR-ENCCs, **(b)** KEGG pathway enrichment analysis of DEGs uniquely identified in TCA-ENCCs. *P*-value and FDR were calculated by clusterProfiler software.

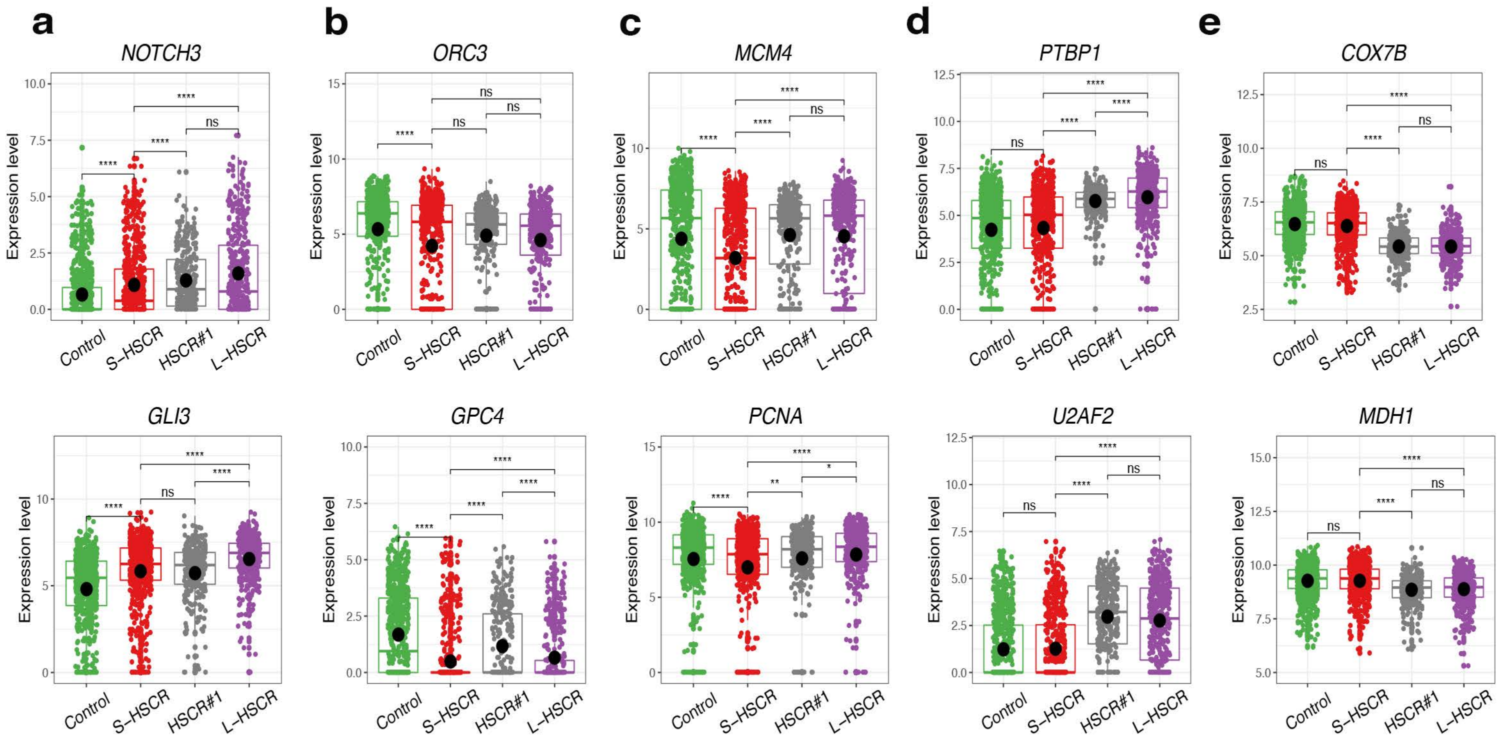

Supplementary Fig. 4 Expressions of representative genes in various cellular pathways. Representative genes of **(a)** negative regulation of neurogenesis, **(b)** synapse organization, **(c)** proliferation, **(d)** RNA splicing and **(e)** energy metabolism. Two-sided Wilcoxon rank-sum test was applied to calculate the significance of difference between two groups. All samples (2 controls with 754 cells, 3 S-HSCR with 643 cells, intermediated HSCR#1 with 306 cells and 2 L-HSCR with 433 cells) were included for comparison and presented as groups. Tests marked by "****", "***", "**" and "*" represent they are statistically different from the controls of *P*-values <0.0001, <0.001, <0.01 and <0.05, respectively. ns, not significantly different. In all the boxplots, each box represents the interquartile range (showing median, 25th and 75th percentile, and 1.5. the interquartile range).

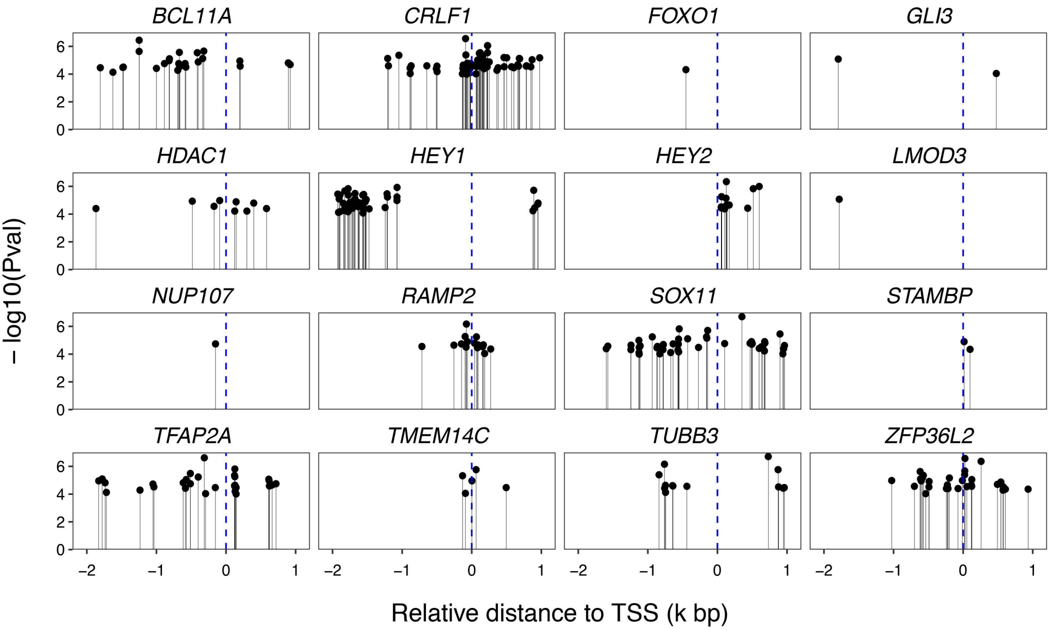

Supplementary Fig. 5 Motif enrichment analysis. Motif enrichment of HDAC1 at the promoter regions (-2k bp to 1k bp relative to TSS) of its target genes. *P*-values (Pval) were calculated by meme FIMO software.

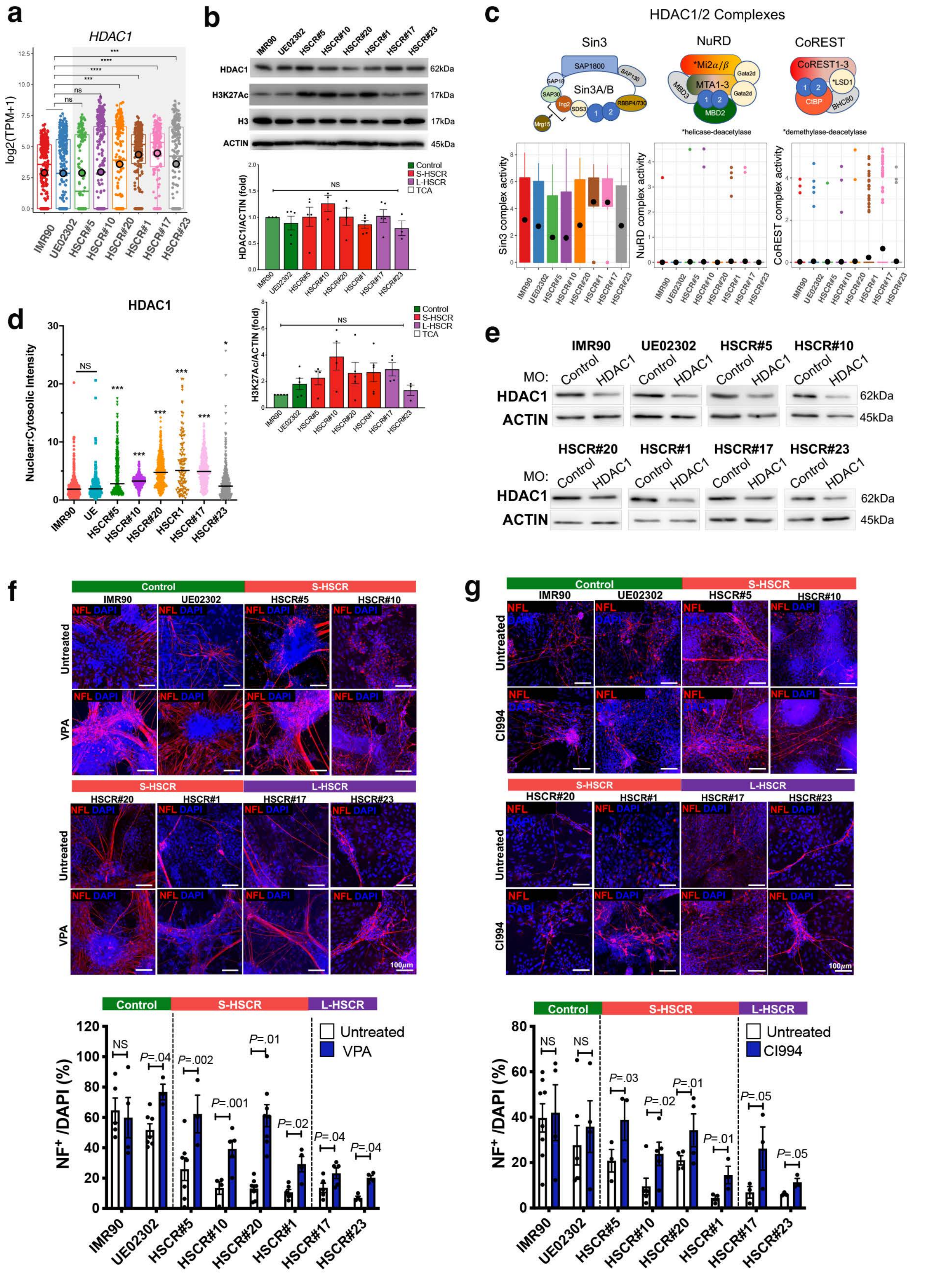

Supplementary Fig. 6 Expression of HDAC1 and its deacetylation activity in control- and HSCR-ENCCs. **(a)** *HDAC1* transcript levels were found to be slightly elevated only in the ENCCs derived from two S-HSCR and two L-HSCR lines as revealed by scRNA-seq analysis. Two-sided Wilcoxon rank-sum test was applied to calculate the significance of difference between two groups (2 controls with 754 cells, 161 HSCR#5 cells, 296 HSCR#10 cells, 186 HSCR#20 cells, 306 HSCR#1 cells, 187 HSCR#17 cells and 246 HSCR#23 cells). Tests marked by "****", "***", "**" and "*" represent they are statistically different from the controls of *P*-values <0.0001, <0.001, <0.01 and <0.05, respectively. ns, not significantly different. In the box plot, each box represents the interquartile range (showing median, 25th and 75th percentile, and 1.5. the interquartile range). **(b)** Western blot analyses show the expression levels of HDAC1 and H3K27Ac in control and HSCR-ENCCs. Representative images are shown. The samples derived from the same experiment and that gels/blots were processed in parallel. The mean expression levels relative to ACTIN from three-independent assays are shown in the bar charts (mean ± SEM). **(c)** The inferred histone deacetylase activities of Sin3, NuRD, CoREST complexes in control and HSCR ENCCs were estimated based on the single-cell gene expression profiles. The mean values are marked by black dots. **(d)** The nuclear and cytoplasmic HDAC1 expression was quantified at the single cell level using CellProfiler. Data show the ratio of nuclear to cytosolic HDAC1 signal in each cell (each dot). The lines represent the median values of nuclear to cytosolic ratio in each group. (control: 226 cells, GW9508:224 cells, SAG: 220 cells, SAG+ETO:226 cells from 4 independent experiments were analyzed). ***, one-way ANOVA test *P*-value < 0.001. **(e)** Western blot shows the expression of HDAC1 in ENCCs treated with control and *HDAC1*-MO. ACTIN is the loading control. Representative images from 3 independent analyses are shown. *In vitro* differentiation assays in absence or presence of HDAC inhibitors: **(f)** Valporic acid (VPA, 1mM) and **(g)** HDAC class I inhibitor (CI994, 500nM). Cells were harvested for immunofluorescence staining at day 5 of neuronal differentiation. Chart shows the mean values +/- SEM from 3-8 independent experiments. (*t*-test, two-sides) Source data are provided as a Source Data file.

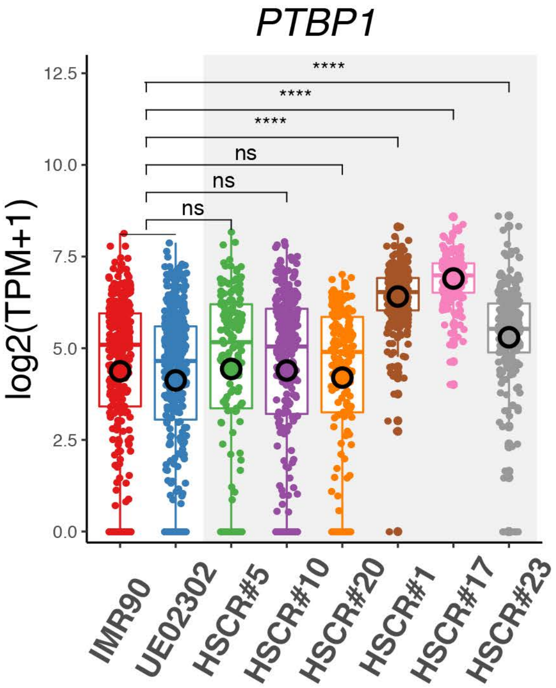

Supplementary Fig. 7 Expression of *PTBP1* in control- and HSCR-ENCCs. Boxplot shows expression level of *PTBP1* in control- and HSCR-ENCCs using scRNAseq data. Two-sided Wilcoxon rank-sum test was applied to calculate the significance of difference between two groups (2 controls with 754 cells, 161 HSCR#5 cells, 296 HSCR#10 cells, 186 HSCR#20 cells, 306 HSCR#1 cells, 187 HSCR#17 cells and 246 HSCR#23 cells). Tests marked by "****", "***", "**" and "*" represent they are statistically different from the controls of *P*-values <0.0001, <0.001, <0.01 and <0.05, respectively. ns, not significantly different. In the box plot, each box represents the interquartile range (IQR, the range between the 25th and 75th percentile) with the mid-point of the data, whiskers indicate the upper and lower value within 1.5 times the IQR.

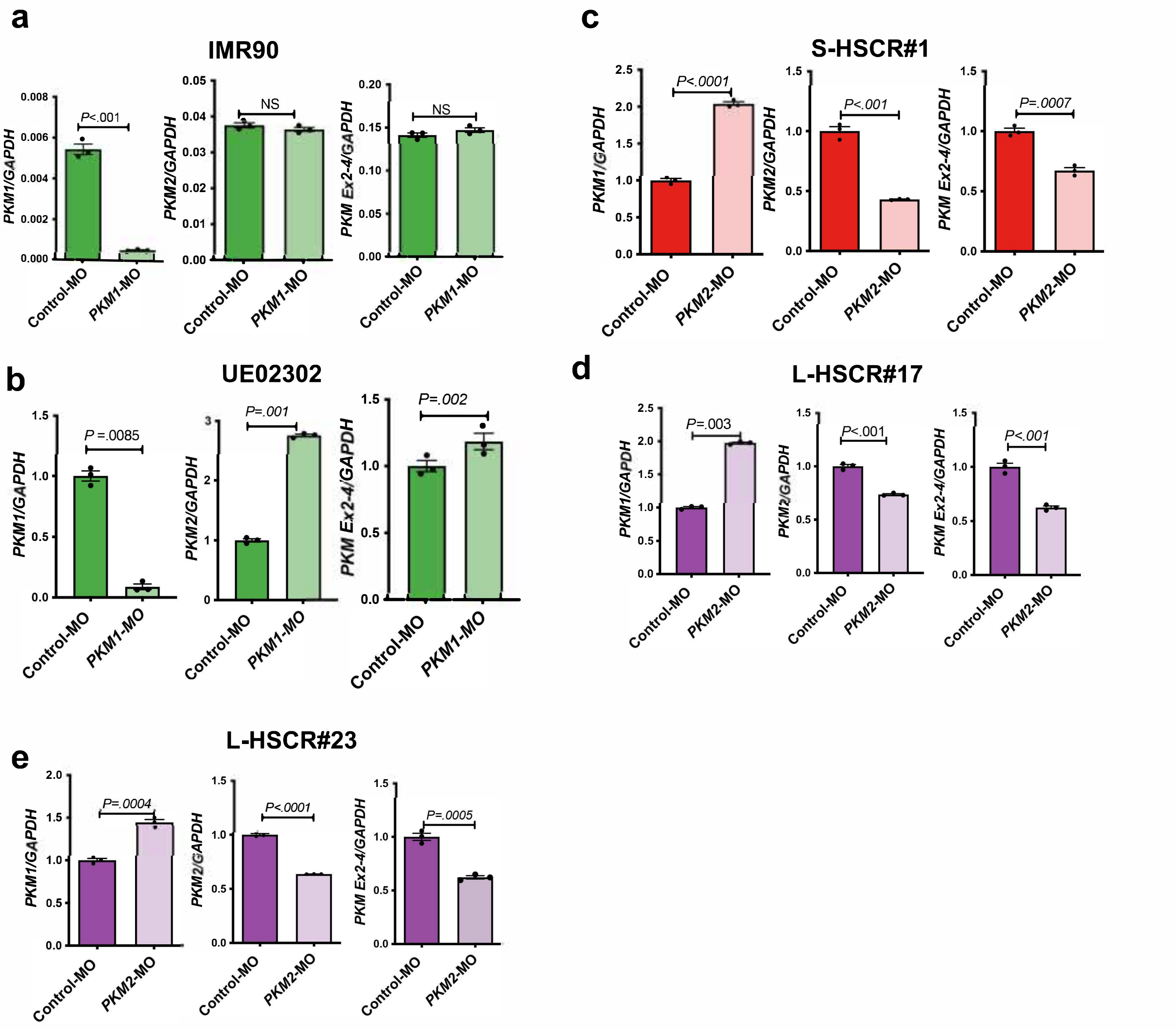

Supplementary Fig. 8 Characterization of morpholinos knockdown cells. RT-qPCR analyses of the expressions of *PKM1, PKM2 and PKM Exon2-4* in control- **(a)** IMR90 & **(b)** UE02302 and HSCR-ENCCs, **(c)** S-HSCR#1, **(d)** L-HSCR#17 & **(e)** L-HSCR-23 transfected with control- and *PKM1*- or *PKM2*-mopholinos (MO). Their expressions relative to *GAPDH* are shown. Data are presented as mean values +/- SEM. (t-test, two-sides)

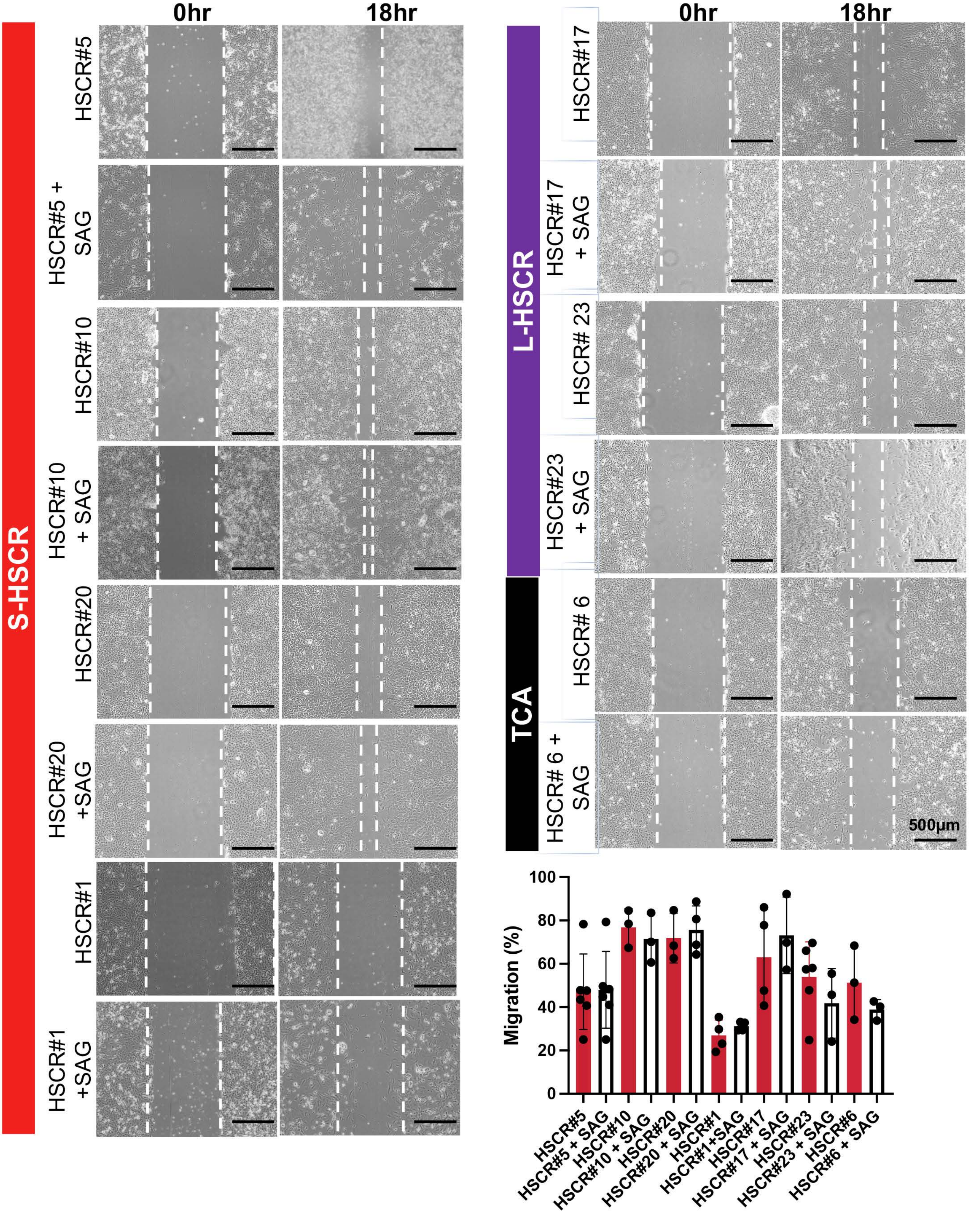

Supplementary Fig. 9 Migration assays with control and SAG primed hENCCs. Representative images of wound healing assays with unprimed and SAG-primed hENCCs. Bar chart shows the quantitative data from 3-8 independent experiments. Data are presented as mean values +/- SEM. (t-test, two-sides). Source data are provided as a Source Data file.

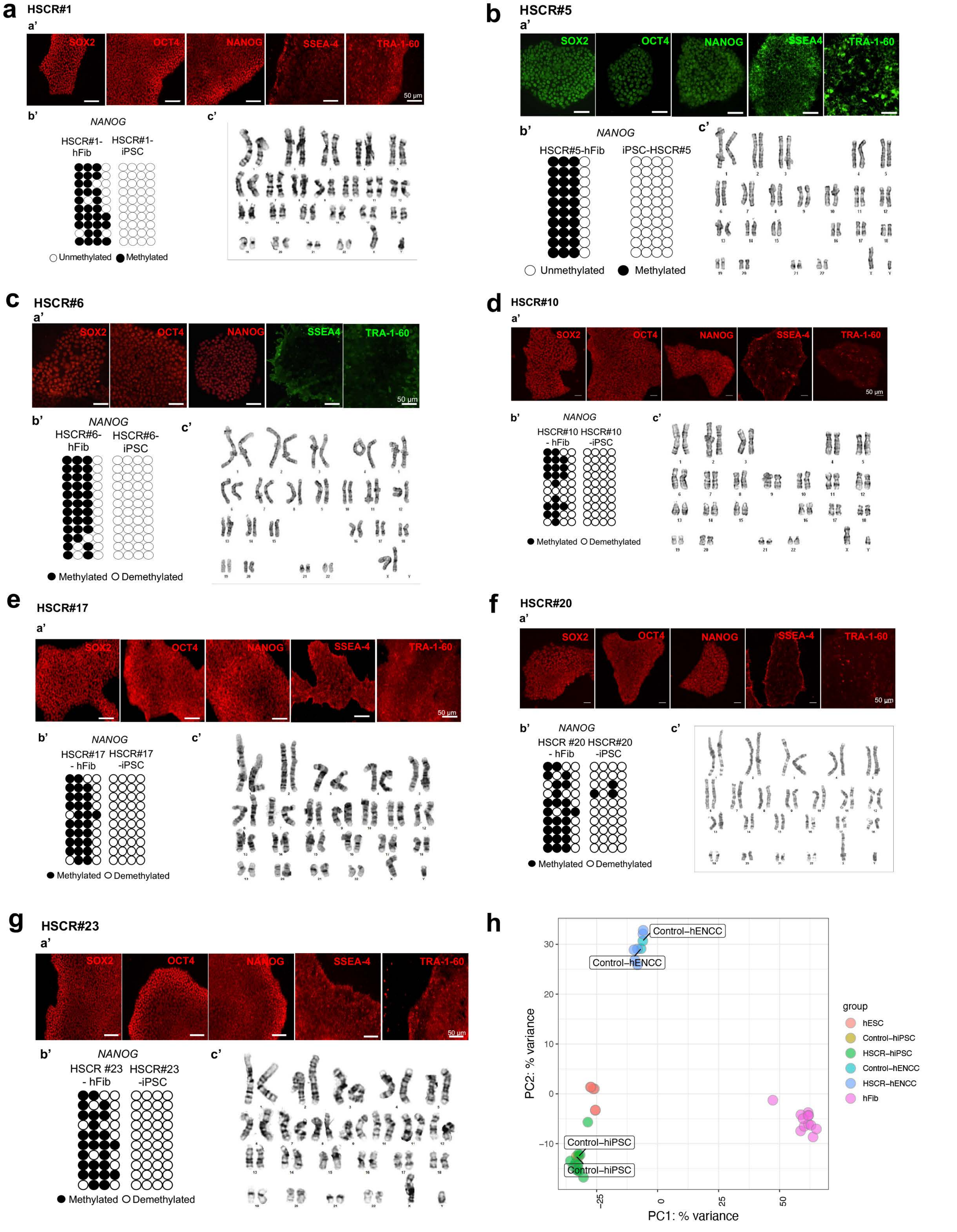

Supplementary Fig. 10 Characterization of HSCR-iPSC lines. a-g: a' Immunocytochemistry analysis on the expression of stem cell markers; **b'** Promoter of *NANGO* is unmethylated; **c'** Normal karyotype in HSCR-iPSC. **h.** PCA analysis of bulk RNA-seq data shows the transcriptomic profiles of all HSCR-iPSC lines are highly comparable to that of hES/hiPSC.

Supplementary Table 1. Primary antibodies used in this study

Primary antibodies	Target antigen	Dilution	Application	Category No.	RRID
Goat-anti-Ret	Receptor tyrosine kinase	1:100	IHC	Neuromics (GT15002)	AB_2179886
Mouse-anti-Tuj1	Neuronal classIII β-tubulin	1:500	IHC	Biolegend (801202)	AB_10063408
Mouse-anti-Sox10 (CL4455)	SOX (SRY-related HMG-box) family transcription factors Sox10	1:100	IHC/ICC	Atlas Antibodies (AMAb91297)	AB_2665884
Goat-anti-Phox2b	Paired-like homeobox 2b	1:200	IHC/ICC	R&D Systems (AF4940)	AB_10889846
Rabbit-anti-Tuj1	Neuronal classIII β-tubulin	1:200	ICC	Abcam (ab18207)	AB_444319
Mouse-anti-Tyrosine Hydroxylase (LNC1)	Tyrosine Hydroxylase	1:200	ICC	Bioscience (MAB318)	AB_2313764
Chicken-anti-Neurofilament-Light (NF)	Neurofilament	1:200	ICC	Neuromics (CH22105)	AB_2737102
Rabbit-anti-PGP9.5	Protein gene product 9.5	1:200	ICC	Abcam (ab108986)	AB_10891773
Mouse-anti-Actin, C4	β-Actin	1:20000	WB	Millipore (MAB1501)	AB_2223041
Anti-HDAC1	HDAC1	1:5000/ 1:500	WB/ICC	Abcam (ab19845)	AB_470299

Anti-Histone H3	Histone 3	1:5000	WB	Abcam (ab4729)	AB_2118291
Rabbit-anti-Phospho-AMPKα (Thr172) (40H9)	p-AMPKα	1:1000	WB	Cell Signaling (2535S)	AB_331250
Rabbit-anti-AMPKα	AMPKα	1:1000	WB	Cell Signaling (2532S)	AB_330331
Rabbit-anti-Phospho-PKM2 (Tyr105)	p-PKM2	1:1000	WB	Cell Signaling (3827S)	AB_1950369
Rabbit-anti-PKM2	PKM2	1:1000	WB	Cell Signaling (3198S)	AB_2252325
Rabbit-anti-Pyruvate Dehydrogenase E1-alpha subunit (phospho S293)	p-PDHA1	1:1000	WB	Abcam (ab177461)	AB_2756339
Pyruvate Dehydrogenase (C54G1)	PDHA1	1:1000	WB	Cell Signaling (3205S)	AB_2162926
CD271-APC	Neurotrophin receptor $p75^{NTR}$	$0.2\mu/1x10^6$ cells	Flow cytometry	Miltenyi Biotec (130110078)	AB_2656849
CD271-FITC	Neurotrophin receptor $p75^{NTR}$	$0.2\mu/1x10^6$ cells	Flow cytometry	Miltenyi Biotec (130091917)	AB_871651
HNK1-APC	CD57	$0.2\mu/1x10^6$ cells	Flow cytometry	BD Biosciences (560845)	AB_10563760
RET-PE	RET	$5\mu/1x10^6$ cells	Flow cytometry	Neuromics (FC15018)	AB_1622005

Supplementary Table 2. Secondary antibodies used in this study

Secondary antibody	Dilution	Application	Category No.	RRID
Alexa Fluor® 488 Donkey-anti-rabbit IgG (H+L)	1:200	IHC	Invitrogen (A21206)	AB_2535792
Alexa Fluor® 488 Donkey-anti-mouse IgG (H+L)	1:200	IHC	Invitrogen (A21202)	AB_141607
Alexa Fluor® 594 Donkey-anti-rabbit IgG (H+L)	1:200	IHC	Invitrogen (A21207)	AB_141637
Alexa Fluor® 594 Donkey-anti-mouse IgG (H+L)	1:200	IHC	Invitrogen (A21203)	AB_141633
Alexa Fluor® 647 Donkey-anti-mouse IgG (H+L)	1:200	IHC	Invitrogen (A31571)	AB_162542
Alexa Fluor® 488 Donkey-anti-guinea pig IgG (H+L)	1:200	IHC	Sigma (SAB4600033)	AB_2890881
Goat-anti-rabbit HRP	1:2000	WB	Dako Cytomation (P0448)	AB_2617138
Goat-anti-mouse HRP	1:2000	WB	Dako Cytomation (P0447)	AB_2617137
Rabbit-anti-goat HRP	1:2000	WB	Dako Cytomation (P0449)	AB_2617143

Supplementary Table 3: List of Morpholinos used in this study.

**Morpholino**	**Target gene**	**Oligo sequences (5'→ 3')**	**Remarks**
Control-MO	-	CCTCTTACCTCAGTTACAATTTATA	
PKM1-MO	*PKM*	CACGAGCTATCTGTAAGGTTTAGGG	Targeting junction between intron8 and exon9
PKM2-MO	*PKM*	GCTGCCGCCTCCTACCTGCCAG	Targeting junction between exon10 and intron10
HDAC1-MO	*HDAC1*	TCGCCTCCCGTCCCTACCGTCAG	Translation blocking

Supplementary Table 4: List of oligos used in this study.

Target gene	Oligo sequences	Annealing Temp (°C)	Product Size (bp)	Experiment used
PKM Ex2-4	Forward: 5'--3' CGGAACACTGGCATCATCTG<br>Reverse: 5'--3' TCTTGATGGTCTCCGCATGG	57.2	142	RT-qPCR
PKM Ex9	Forward: 5'--3' CAGCCAAAGGGGACTATCCT<br>Reverse: 5'--3' GAGGCTCGCACAAGTTCTTC	56.5	99	RT-qPCR
PKM Ex10	Forward: 5'--3' CTATCCTCTGGAGGCTGTGC<br>Reverse: 5'--3' GTGGGGTCGCTGGTAATG	57.2	133	RT-qPCR
PTBP1 ex9	Forward: 5'--3' GGTTTCCCTCCCACCTTTGCC<br>Reverse: 5'--3' GGTGTGACTCTCTCTGGGTTGAGG	60	197	RT-qPCR
GAPDH	Forward: 5'--3' CAAGAAGGTGGTGAAGCAGGC<br>Reverse: 5'--3' GCCAAATTCGTTGTCATACCAGGA	60	184	RT-qPCR
