# Supplementary material for: Transcriptomics of Hirschsprung disease patient-derived enteric neural crest cells reveals a role for oxidative phosphorylation
Source: Nat Commun. 2023 Apr 15;14:2157. doi: 10.1038/s41467-023-37928-5 (PMC10105741; doi:10.1038/s41467-023-37928-5)
Supplement: Supplementary file 3 — Reporting Summary [file 41467_2023_37928_MOESM3_ESM.pdf]

nature portfolio

Corresponding author(s): Elly Sau-Wai NGAN

Last updated by author(s): Apr 5, 2022

Reporting Summary

Nature Portfolio wishes to improve the reproducibility of the work that we publish. This form provides structure for consistency and transparency in reporting. For further information on Nature Portfolio policies, see our Editorial Policies and the Editorial Policy Checklist.

Statistics

For all statistical analyses, confirm that the following items are present in the figure legend, table legend, main text, or Methods section.

n/a	Confirmed	
☐	☒	The exact sample size (*n*) for each experimental group/condition, given as a discrete number and unit of measurement
☐	☒	A statement on whether measurements were taken from distinct samples or whether the same sample was measured repeatedly
☐	☒	The statistical test(s) used AND whether they are one- or two-sided<br>*Only common tests should be described solely by name; describe more complex techniques in the Methods section.*
☐	☒	A description of all covariates tested
☐	☒	A description of any assumptions or corrections, such as tests of normality and adjustment for multiple comparisons
☐	☒	A full description of the statistical parameters including central tendency (e.g. means) or other basic estimates (e.g. regression coefficient) AND variation (e.g. standard deviation) or associated estimates of uncertainty (e.g. confidence intervals)
☐	☒	For null hypothesis testing, the test statistic (e.g. *F*, *t*, *r*) with confidence intervals, effect sizes, degrees of freedom and *P* value noted<br>*Give P values as exact values whenever suitable.*
☒	☐	For Bayesian analysis, information on the choice of priors and Markov chain Monte Carlo settings
☒	☐	For hierarchical and complex designs, identification of the appropriate level for tests and full reporting of outcomes
☐	☒	Estimates of effect sizes (e.g. Cohen's *d*, Pearson's *r*), indicating how they were calculated

Our web collection on statistics for biologists contains articles on many of the points above.

Software and code

Policy information about availability of computer code

Data collection	N.A
Data analysis	The FlowJo (version 8.2), GraphPad Prism (version 9), R (version 3.6.2), R package Seurat (version 3.1.4), Cutadapt (version 1.7), Bowtie2 (version 2.3.4.1), STAR (version 2.7.0), FastQC (version 0.11.6), RSEM (version 1.3.0), monocle (version 2.14.0), clusterProfiler (version 3.14.3), SCENIC (version 1.1.2), MEME (version 5.4.1), SUPPA (version 2.3), Cytoscape (version 3.7.2), GraphPad Prism (version 9) were included for data analysis in this research.<br>Custom codes: https://github.com/ellylab/HSCR-scRNAseq-paper or https://doi.org/10.5281/zenodo.7710574

For manuscripts utilizing custom algorithms or software that are central to the research but not yet described in published literature, software must be made available to editors and reviewers. We strongly encourage code deposition in a community repository (e.g. GitHub). See the Nature Portfolio guidelines for submitting code & software for further information.

Data

Policy information about availability of data

All manuscripts must include a data availability statement. This statement should provide the following information, where applicable:

- Accession codes, unique identifiers, or web links for publicly available datasets
- A description of any restrictions on data availability
- For clinical datasets or third party data, please ensure that the statement adheres to our policy

Raw sequencing data are available in the Sequence Read Archive (SRA) at the NCBI Center with the accession number PRJNA784249 (including 1 mouse cardiac NCCs and 9 human iPSC-derived BioSamples). The processed scRNA-seq data sets are available at https://doi.org/10.5281/zenodo.6104610. Public datasets that used for comparison in this article can be downloaded from https://doi.org/10.5281/zenodo.5588286 (mouse enteric NCCs, Fig. 1k), http://

atlas.gs.washington.edu/mouse-rna (mouse NCCs on enteric and peripheral nervous system, Fig. 1k) and GSE149524 (ENS cells at E15.5, E18.5 and P21). Reference ENSEMBL GRCh38 (release 90) can be downloaded from https://ftp.ensembl.org/pub/release-90/fasta/homo_sapiens/. A reporting summary for this Article is available as a Supplementary Information file. Source data are provided with this paper.

Field-specific reporting

Please select the one below that is the best fit for your research. If you are not sure, read the appropriate sections before making your selection.

☒ Life sciences ☐ Behavioural & social sciences ☐ Ecological, evolutionary & environmental sciences

For a reference copy of the document with all sections, see nature.com/documents/nr-reporting-summary-flat.pdf

Life sciences study design

All studies must disclose on these points even when the disclosure is negative.

Sample size	Seven patients with HSCR from different subgroups, including 4 S-HSCR, 2 L-HSCR and 1TC, were recruited for this study. The sample size of each group was determined based on the incidence of the each subtypes (S-HSCR>L-HSCR>TCA).<br>For all the biological assays, at least three independent experiments were performed with multiple replicates. The replicate number and sample size were determined based on the assay type and the difference between groups (control vs HSCR or untreated vs treatment groups). We considered the sample sizes to be sufficient when low variability between samples was observed.
Data exclusions	No data was excluded.
Replication	Each experiment in this study was independently repeated for at least 3 times to generate final conclusion. The replication of experiments showed similar results or trends.
Randomization	All the human specimens and organisms involved in this study were allocated in randomization.
Blinding	Blinding was performed in data collection stage of scRNA-seq. Cellular and molecular experiments were not performed in blind, since analysis were performed under selectively grouping and conditions.

Reporting for specific materials, systems and methods

We require information from authors about some types of materials, experimental systems and methods used in many studies. Here, indicate whether each material, system or method listed is relevant to your study. If you are not sure if a list item applies to your research, read the appropriate section before selecting a response.

Materials & experimental systems

n/a	Involved in the study	
☐	☒	Antibodies
☐	☒	Eukaryotic cell lines
☒	☐	Palaeontology and archaeology
☒	☐	Animals and other organisms
☐	☒	Human research participants
☒	☐	Clinical data
☒	☐	Dual use research of concern

Methods

n/a	Involved in the study	
☒	☐	ChIP-seq
☐	☒	Flow cytometry
☒	☐	MRI-based neuroimaging

Antibodies

Antibodies used

For flow cytometry:
CD271-APC Neurotrophin receptor 0.2μ/1x106 cells Flow cytometry Miltenyi Biotec (130110078) AB_2656849
CD271-FITC Neurotrophin receptor 0.2μ/1x106 cells Flow cytometry Miltenyi Biotec (130091917) AB_871651
HNK1-APC 0.2μ/1x106 cells Flow cytometry BD Biosciences (560845) AB_10563760
RET-PE 5[illegible]/1x106 cells Flow cytometry Neuromics (FC15018) AB_1622005

For western blot:
Mouse-anti-Actin β-Actin, clone C4, 1:20000 WB Millipore (MAB1501) AB_2223041
Rabbit-anti-HDAC1 1:5000/1:500 WB/ICC Abcam (ab19845) AB_470299
Rabbit-anti-Histone H3 Histone 3 1:5000 WB Abcam (ab4729) AB_2118291
Rabbit-anti-Phospho-AMPKα (Thr172) (40H9) 1:1000 WB Cell Signaling (2535S) AB_331250
Rabbit-anti-AMPKα 1:1000 WB Cell Signaling (2532S) AB_330331
Rabbit-anti-Phospho-PKM2 (Tyr105) 1:1000 WB Cell Signaling (3827S) AB_1950369
Rabbit-anti-PKM2 1:1000 WB Cell Signaling (3198S) AB_2252325
Rabbit-anti-Pyruvate Dehydrogenase E1-alpha subunit (phospho S293) 1:1000 WB Abcam (ab177461) AB_2756339
Rabbit-anti-Pyruvate Dehydrogenase (C54G1) 1:1000 WB Cell Signaling (3205S) AB_2162926

Goat-anti-rabbit HRP 1:2000 WB Dako Cytomation(P0448) AB_2617138
Goat-anti-mouse HRP 1:2000 WB Dako Cytomation (P0447) AB_2617137
Rabbit-anti-goat HRP 1:2000 WB Dako Cytomation (P0449) AB_2617143

For ICC:
Mouse-anti-Sox10 (CL4455) 1:100 IHC/ICC Atlas Antibodies (AMAb91297) AB_2665884
Rabbit-anti-Tuj1 1:200 ICC Abcam (ab18207) AB_444319
Mouse-anti-Tyrosine Hydroxylase (LNC1) 1:200 ICC Bioscience (MAB318) AB_2313764
Chicken-anti-Neurofilament-Light (NF) 1:200 ICC Neuromics (CH22105) AB_2737102
Rabbit-anti-PGP9.5 Protein gene product 9.5 1:200 ICC Abcam (ab108986) AB_10891773
Alexa Fluor® 488 Donkey-anti-rabbit IgG (H+L) 1:200 IHC Invitrogen (A21206) AB_2535792
Alexa Fluor® 488 Donkey-anti-mouse IgG (H+L) 1:200 IHC Invitrogen (A21202) AB_141607
Alexa Fluor® 594 Donkey-anti-rabbit IgG (H+L) 1:200 IHC Invitrogen (A21207) AB_141637
Alexa Fluor® 594 Donkey-anti-mouse IgG (H+L) 1:200 IHC Invitrogen (A21203) AB_141633
Alexa Fluor® 647 Donkey-anti-mouse IgG (H+L) 1:200 IHC Invitrogen (A31571) AB_162542
Alexa Fluor® 488 Donkey-anti-guinea pig IgG (H+L) 1:200 IHC Sigma (SAB4600033) AB_2890881

Validation

All antibodies used in this study were obtained from commercial sources, and validations of all the primary antibodies for the use in human cells and for the flow cytometry, Western blotting and/or ICC analyses are available in manufactures' website.

Eukaryotic cell lines

Policy information about cell lines

Cell line source(s)

IMR90-iPSC, WiCell Research Resources (WiCell, WI., RRID:CVCL C434).
UE02302-iPSC, was generated from urine derived cells of a male individual by episomal reprogramming vectors carrying the four reprogramming factors (Xue, Y. et al. PLoS One 8, e70573 (2013).
Seven HSCR-iPSC lines were generated in this study including (four S-HSCR-iPSC,:HSCR#5, #10, #20, #1; L-HSCR: HSCR#17, HSCR#23; TCA: HSCR#6)

Authentication

All the iPSC lines generated in this study were fully characterized following the standard protocols including ICC with pluripotent stem cell markers (SOX2, OCT4, NANGO, SSEA-4, TRA-1-6), methylation marks in NANOG promoter, Karyotyping and bulk RNAseq.

Mycoplasma contamination

All the iPSC lines were tested negative for mycoplasma contamination.

Commonly misidentified lines
(See ICLAC register)

No commonly misidentified line was involved in this study.

Human research participants

Policy information about studies involving human research participants

Population characteristics

Seven HSCR patients from different subgroups (4 S-HSCR, 2 L-HSCR and 1 TCA) were recruited with a male to female predominance of 6:1. Two control iPSC lines from both genders were included. Each patient was given a disease score according to the extent of aganglionsis (0-5; control= 0 and TCA=5). The RET alleles in rs2435357 and variants in the coding region of RET gene were identified through sequencing the genomic DNA from the patients' blood. The gender and genotype of each patient was confirmed by transcriptomic analyses and Sanger sequencing, respectively. Given that a small sample size was included, no sex- and gender-based analyses have been performed.

Recruitment

All the patients were recruited at Queen Mary hospital, Hong Kong. Samples from four S-HSCR, 2 L-HSCR and 1 TCA patients were included in this study based on the incidence of the subtypes (S-HSCR>L-HSCR>TCA). Only patients who did not carry any known HSCR associated coding region mutation were included in this study to define the common factors associated with the HSCR patients with unknown etiology. Informed consent was obtained by participants.

Ethics oversight

The study was approved by the institutional review board of The University of Hong Kong together with the Hospital Authority (UW 13-419).

Note that full information on the approval of the study protocol must also be provided in the manuscript.

Flow Cytometry

Plots

Confirm that:

☒ The axis labels state the marker and fluorochrome used (e.g. CD4-FITC).

☒ The axis scales are clearly visible. Include numbers along axes only for bottom left plot of group (a 'group' is an analysis of identical markers).

☒ All plots are contour plots with outliers or pseudocolor plots.

☒ A numerical value for number of cells or percentage (with statistics) is provided.

Methodology

Sample preparation	The sample preparation procedures for flow cytometry or FACS were described in Material and Methods section.
Instrument	The labeled cells were detected using FACSAriaIII (Becton Dickinson Immunocytometry Systems, San Jose, CA, USA). Isotype-matched antibodies were used as controls.
Software	FlowJo version 8.2 (Tree Star, Inc.) was used to analyze flow data.
Cell population abundance	The population abundances of HNK+/p75NTR+ and RET+ cells were included in Supplementary Fig. 1a and 1c.
Gating strategy	IgG control was used as the negative control for gating setting as shown in Supplementary Fig. 1a and 1c.

☒ Tick this box to confirm that a figure exemplifying the gating strategy is provided in the Supplementary Information.
