# Supplementary material for: Transcriptomics of Hirschsprung disease patient-derived enteric neural crest cells reveals a role for oxidative phosphorylation
Source: Nat Commun. 2023 Apr 15;14:2157. doi: 10.1038/s41467-023-37928-5 (PMC10105741; doi:10.1038/s41467-023-37928-5)
Supplement: Supplementary file 4 — Description of Additional Supplementary Files [file 41467_2023_37928_MOESM4_ESM.pdf]

Description of Additional Supplementary Files

File Name: Supplementary Data 1
Description: Sequencing information of individual samples.

File Name: Supplementary Data 2
Description: The expression dynamics of 833 top DEGs were catalogued into 4 major modules.

File Name: Supplementary Data 3
Description: Top AS events contributed to PC2 in Fig. 4e
